# Novel Compound Heterozygous Mutation of the *ABCA3* Gene in a Patient with Neonatal-Onset Interstitial Lung Disease

**DOI:** 10.3390/jcm14113704

**Published:** 2025-05-25

**Authors:** Gregorio Serra, Veronica Notarbartolo, Vincenzo Antona, Caterina Cacace, Maria Rita Di Pace, Daniela Mariarosa Morreale, Marco Pensabene, Ettore Piro, Ingrid Anne Mandy Schierz, Maria Sergio, Giuseppina Valenti, Mario Giuffrè, Giovanni Corsello

**Affiliations:** 1 Department of Health Promotion, Mother and Child Care, Internal Medicine and Medical Specialties “G. D’Alessandro”, University of Palermo, 90133 Palermo, Italy; gregorio.serra@unipa.it (G.S.); vincenzo.antona@policlinico.pa.it (V.A.); mariarita.dipace@policlinico.pa.it (M.R.D.P.); danymorreale97@gmail.com (D.M.M.); marco.pensabene@policlinico.pa.it (M.P.); ettore.piro@unipa.it (E.P.); ingridannemandy.schierz@policlinico.pa.it (I.A.M.S.); maria.sergio@policlinico.pa.it (M.S.); giusi.valenti.1997@gmail.com (G.V.); mario.giuffre@unipa.it (M.G.); giovanni.corsello@unipa.it (G.C.); 2 Neonatal Intensive Care Unit, “Barone Ignazio Romeo” Hospital, 98066 Patti, Italy; cacacecaterina@gmail.com

**Keywords:** chILDs, interstitial disease, newborn, surfactant, respiratory insufficiency

## Abstract

**Background:** Children’s interstitial and diffuse lung diseases, commonly referred to as “chILDs”, include around 200 rare conditions that disrupt normal lung function. They are classified, based on etiopathogenesis, into several subgroups, having a varied and multifaceted clinical presentation depending on the type of genetic mutation present. **Methods and Results:** We describe the case of a late preterm newborn presenting soon after birth with respiratory distress syndrome poorly responsive to surfactant administration, in whom a targeted gene panel analysis for pulmonary congenital diseases, performed using next-generation sequencing (NGS), revealed a novel compound heterozygous variant of the ATP-Binding-Cassette-Subfamily-A-Member-3 (ABCA3) gene. A review of the literature on the subject completes our work. **Conclusions:** Molecular genetic analysis has become crucial for a more targeted therapeutic treatment, along with the only current curative treatment option that is lung transplantation.

## 1. Introduction

Children’s interstitial and diffuse lung diseases (also known as “chILDs”) encompass about 200 rare conditions impairing lung function. They manifest with non-specific symptoms, including coughing, dyspnea, and respiratory insufficiency. Gene transmission is described as both autosomal recessive and dominant [1]. They are classified, based on etiopathogenesis, into different subgroups: diffuse developmental disorders, growth abnormalities, structural changes in the lung associated with chromosomal anomalies, specific conditions of undefined etiology, surfactant dysfunction, and other conditions related to surfactant dysfunction in the absence of a known genetic cause [2]. Diagnosis is made with clinical pictures, radiological investigations (i.e., chest X-ray and computed tomography [CT] scan), alveolar bronchoscopy, lung biopsy with histological examination, and, recently, genetic tests, including next-generation sequencing (NGS), which are increasingly used and crucial in clinical practice [3,4]. Currently, no established and effective treatments are available for these rare disorders, with the exception of lung transplantation [1,2]. The latter, however, is burdened by major comorbidities, difficulties in finding compatible donors, and high mortality rates. Research is underway to investigate potential new and promising therapeutic tools, like gene therapy [1,2]. We describe the case of a late preterm newborn presenting right after birth with respiratory distress syndrome poorly responsive to surfactant administration, in whom a targeted gene panel analysis, performed using NGS, revealed a novel compound heterozygous variant of the ATP-Binding-Cassette-Subfamily-A-Member-3 (*ABCA3*) gene. In addition, a bioinformatic prediction analysis on the protein function of the identified variants was performed. Finally, a review of previously reported patients carrying homozygous or compound heterozygous variants of the same gene was conducted. We focused on commonalities and differences among them, analyzing clinical manifestations, therapies administered, and outcomes. The aim of the present report is to provide a better clinical and genomic characterization of these rare diseases, contributing to enriching the current mutational spectrum of such conditions and to future research on new effective therapeutic approaches.

## 2. Case Report and Literature Review

Our proband is a female late preterm neonate born from vaginal delivery at 35^+5^ weeks of gestation due to chorioamnionitis to healthy, nonconsanguineous parents. She is the first child of the couple. The family history was unremarkable, including two healthy consanguineous sisters of the proband, daughters of the same father and a different mother.

**Figure 1:**
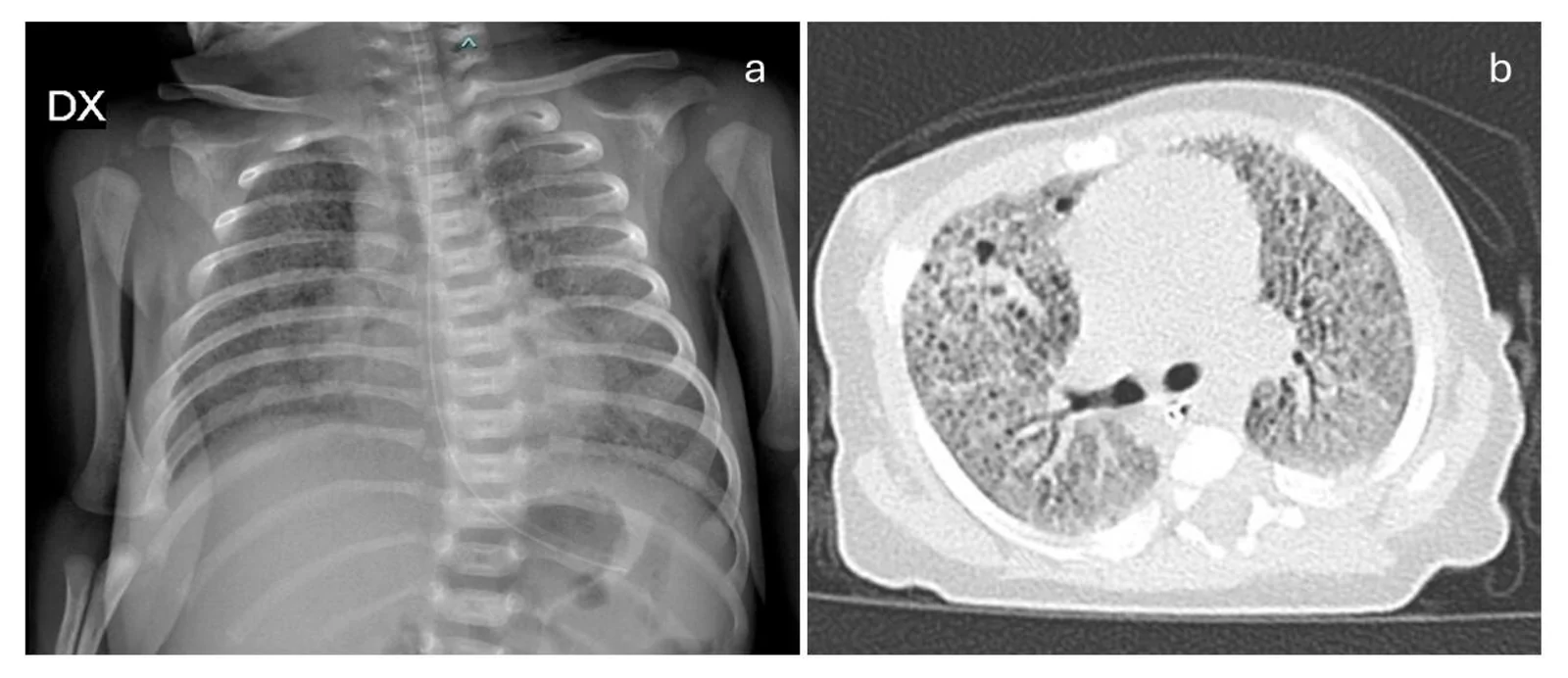
(**a**) Chest X-ray shows hypodiaphany in all lung areas, in addition to texture reinforcement with a reticulo-micronodular pattern; (**b**) Chest CT: in both lungs, a diffuse increase in parenchymal density is observed, with a predominantly “ground glass” appearance and associated thickening of the inter- and intralobular septa. Bilateral, multiple, subcentimeter-sized air-filled cavities are present.

During pregnancy, the mother reported to be a tobacco user, smoking 4–5 cigarettes/day. The proband’s birth occurred at a level I hospital in a small town in Eastern Sicily, neighboring the urban city of Palermo, the regional capital. Apgar scores were 7 and 9 at 1 and 5 min, respectively. Anthropometric measures were as follows: weight 2270 g (79th centile, SDS −0.47), length 46 cm (48th centile, SDS −0.06), head circumference 33 cm (74th centile, SDS 0.64), according to the Italian INeS Growth Charts [5]. Postnatally, due to respiratory distress and oxygen dependence, which had been present since birth, the baby was transferred through the neonatal emergency transport service to the Neonatal Intensive Care Unit (NICU) closest to the birth center. There, her respiratory function gradually worsened, so she was intubated and treated with exogenous surfactant administration. However, after an initial mild improvement, which allowed extubation, she needed non-invasive ventilatory support. Therefore, for the emerging suspicion of congenital respiratory disease, she was transferred, at around two weeks of life, to our NICU, located at the Mother and Child Department of the University Hospital of Palermo, to continue both diagnostic path and therapeutic management. During hospitalization in our unit, non-invasive respiratory assistance was provided, with a gradual increase in oxygen requirement up to 100%. Three doses of surfactant were also administered through the less invasive surfactant administration (LISA) technique, giving only partial and short benefits. The following clinical course was further marked by two episodes of severe and life-threatening blood infections caused by *Serratia marcescens* and *Klebsiella pneumoniae* (proven by blood culture), from which our patient slowly recovered after specific antibiotic therapy (amikacin and meropenem, lasting 14 and 10 days, respectively). At blood gas monitoring, a tendency to hypercapnia (with pH within the normal range, through the metabolic compensation obtained with the increase in bicarbonate levels) was observed, which slowly and gradually normalized after the first month of life. Meanwhile, laboratory tests revealed neutrophilic leukocytosis, elevated inflammatory markers, and cytolysis indexes (lactate dehydrogenase, LDH, 738 IU/L; normal values < 435), with normal hepatic and renal function tests. Neurological examination showed a generalized central type hypotonia, with normal osteotendinous and primitive reflexes. A brain ultrasound (US) revealed findings compatible with previous hypoxic-ischemic episodes (millimetric cavitated lesions of the germinal matrix of the left lateral ventricle). Abdominal US scans and ophthalmological evaluation showed no abnormalities, while echocardiography disclosed an interatrial defect, and then a mild-to-moderate pulmonary hypertension for which a diuretic treatment with furosemide, spironolactone, and hydrochlorothiazide, in addition to sildenafil, was required. Then, she underwent a chest X-ray and CT scans, which showed a lung picture with ‘ground glass’ appearance, compatible with congenital surfactant protein deficiency (Figure 1a,b).

NGS analysis of a panel of genes involved in congenital lung diseases detected the c.464 G>A and c.2921G>A variants, in compound heterozygosity, in the *ABCA3* gene (for details, see the Section 2.1). Given the results of the genetic test, the diagnosis of congenital surfactant protein deficiency was made. It was not possible to test the ABCA3 protein expression in the lung, as the parents refused any further invasive investigation on their daughter, including either biopsy or the dosage of any of the surfactant proteins (SPA-SPD) in the bronchoalveolar lavage fluid (and also in the blood, also due to difficulties linked to the unavailability of a dedicated laboratory in either our center or region). Thereafter, the most recently available treatment protocol for childhood interstitial lung disease (European protocols for the diagnosis and initial treatment of interstitial lung disease in children, 2015 [6]) was started at 2 months of life. Before, it included the administration of intravenous steroids (10 mg/kg per day progressively decreased up to 2.5 mg/kg/day within one week) and then oral ones (methylprednisolone 1mg/kg/die, and every other day for the last 3 days) for an overall 14-day course, as well as oral azithromycin (10 mg/kg every other day, for 11 weeks) and, subsequently (at age of 3 months), hydroxychloroquine (10 mg/kg/day).

During the first trimester of hospitalization, the baby was mainly fed through parenteral nutrition despite effective suction, with the eventual support of high-caloric formula, to reduce energy expenditure. Later, at age 3 months, in light of frequent episodes of desaturation during feeding and the increased risk of *ab ingestis* pneumonia, a percutaneous endoscopic gastrostomy (PEG) was surgically placed. After about a 4-month hospitalization in the NICU, where specific physyokinesis and logopedic treatments were also provided, and having reached cardio-respiratory stability, she was allowed to stay within a rooming-in setting with her parents. There, with the support of the whole care team, including periodic psychological sessions, they progressively acquired the skills needed for home management. Her condition remained stable, the ventilatory support was weaned to high-flow oxygen therapy with an average fraction of inspired oxygen requirement of 45%, and she showed slow, mild but constant growth through PEG feeding. Finally, after the complex activation of the home medical-nursing service, she was discharged at around 5 months of age and enrolled in a follow-up program shared between the NICU closest to home (for the management of emergencies) and our outpatient service (for routine longitudinal multidisciplinary evaluations). At 9 months of age (8 months and 13 days of corrected age), our baby was admitted again to our department for the replacement and size refinement of the PEG device. On admission, she was in good clinical general condition, supported through high-flow nasal cannula (40% as a fraction of inspired oxygen requirement). The auxological parameters were as follows: weight 6350 g (2nd centile, SDS −1.98), length 69 cm (45th centile, SDS −0.13), and head circumference 44.5 cm (76th centile, SDS +0.71), according to the World Health Organization growth chart for neonatal and infant close monitoring [7]. She showed axial hypotonia, she was able to maintain the sitting position with support, and a parachute reflex was present both in the latero-lateral and antero-posterior directions. Laboratory tests prior to discharge showed a normal complete blood count, including the leukocyte differential, negative inflammatory markers, and cytolysis indices, as well as hepatic and renal function tests. Chest X-ray and heart US evidenced, as well, no significant variations from previous findings (bilateral reticular thickening of the lung parenchyma and tricuspid insufficiency and mild-moderate pulmonary hypertension, respectively), while a head US identified supratentorial triventricular dilatation, suggestive of a possible evolution of the Grade I intraventricular hemorrhage observed after birth. Auditory evoked potentials (AEPs) were performed, but they were not conclusive due to the interference of the ventilatory support. Nonetheless, transient evoked otoacoustic emissions, as well as the oriented response of the head towards acoustic stimuli as evidenced on neurological assessment, suggested a normal hearing function. Soon after, she was discharged in good general condition, with the indication to continue at home the prescribed therapy, including sildenafil and hydroxychloroquine (0.5 mg/kg 4 times in a day and 10 mg/kg/day, respectively; see above), and with a high-calorie weaning diet designed to promote more regular growth. Currently, at age 1 year, she is continuously supported through high-flow nasal cannula with a stable oxygen requirement of about 40%, and neither further clinical problems nor additional abnormalities disclosed through multiorgan US assessment are documented. The opportunity of a more invasive therapeutic approach with lung transplantation, already discussed at the time of first hospitalization, and at present more achievable, has been proposed to the parents. This will be explained to them in detail within the currently planned forthcoming multidisciplinary assessment by the medical staff of the transplant reference center of our region; they will also establish whether the patient fulfills the eligibility criteria for the procedure. At a telematic meeting, we encountered the parents for genetic counseling, providing them with recurrence risk and management opportunities for future pregnancies, including preconception and prenatal diagnosis. Actually, our team was contacted again a few weeks later, as the mother was within the first trimester of her second gestation. Therefore, we offered the couple a prenatal target NGS analysis of the known familial variants of *ABCA3* on a chorionic villi sample. Such invasive genetic investigation disclosed in the embryo the presence, in homozygosity, of the only c.464G>A variant, inherited from the mother. The c.2921G>A variation was, conversely, absent. Therefore, the analysis of DNA polymorphisms (Short Tandem Repeats, STR-microsatellites) was performed, which evidenced a maternal uniparental isodisomy of chromosome 16 (see Supplementary File S1). Such an epigenetic mechanism explained the likely, although rare [8], process subtending the genetic profile of the embryo (the homozygous c.464G>A variant in the *ABCA3* gene), eventually leading the couple to end the pregnancy.

### 2.1. Genetic Analysis

DNA was extracted from the peripheral blood of the proband and parents. A panel sequencing analysis was performed. It focused on the coding regions and exon-intron junctions (±5 bp) of genes related to the clinical suspicion, i.e., the suspected surfactant protein deficiency, including several genes, in addition to *ABCA3*, which are summarized in Supplementary File S2.

NGS sequencing was carried out *in trio* with a ClinEX pro kit (4bases) on the NovaSeq6000 platform (Illumina Inc., San Diego, CA, USA). Analytical sensitivity and specificity were >99%. Sequence analysis detected the c.464 G>A and c.2921G>A variants, in compound heterozygosity, in the *ABCA3* gene. Such variants are responsible, at the protein level, for the p.Arg155Gln and p.Gly974Glu amino acid changes, respectively (rs1201230394). The c.464 G>A variant, inherited from the mother, is not present in the general population allele frequency database (gnomAD) and is described in the scientific literature as associated with surfactant protein deficiency when identified either in compound heterozygosity or homozygosity [9]. It is classified according to the guidelines of the American College of Medical Genetics and Genomics (ACMG [10]) as likely pathogenic (class 4). The c.2921G>A variant, transmitted by the father, is likewise not present in the general population polymorphism database (gnomAD), but conversely, it is not reported in the scientific literature. This latter is classified as a variant of uncertain significance (VUS, class 3), according to ACMG [10]. Next-generation sequencing analysis to search for the paternal c.2921G>A variant of the *ABCA3* gene was eventually performed on both consanguineous sisters, in whom, however, it was absent.

Bioinformatic analysis was performed using the BWA Aligner/DRAGEN Germline Pipeline/DRAGEN Enrichment systems (Illumina Inc., San Diego, CA, USA). Sequences were aligned to the human reference genome GRCh37. Geneyx Analysis software latest Version 6.1 (Knowledge-Driven NGS analysis tool powered by the GeneCards Suite, Rehovot, Israel) was used to filter and prioritize variants. HPO, OMIM, and/or Gene Reviews databases were consulted to select genes associated with the clinical indication. Only variants in the selected genes, with appropriate read depth and quality parameters, were considered [11]. Variants were annotated according to the HGVS nomenclature and classified according to the ACMG standard guidelines [10,12]. For the interpretation of variants, our laboratory referred to the scientific literature, ClinVar, HGMD, LOVD databases, and gene and/or disease-specific databases; for the allelic frequency, we referred to gnomAD v2.1.1 population database and to the internal database of the current laboratory. The confirmation test was performed on a second DNA extraction. The laboratory where the analysis was performed is certified according to the reference standard UNI EN ISO 9001.2015 [13] and participates in the external quality controls GenQA (Genomics Quality Assessment).

### 2.2. Bioinformatic Analysis on Protein Function of Identified Variants

For bioinformatic analysis of the identified variants, tools such as SIFT [14], PolyPhen-2 [15], MutationTaster [16], and Panther [17] were used to predict their impact on protein function. The first variant (maternal allele), c.464G>A, leading to the substitution p.Arg155Gln (R155Q), and the second one (paternal allele), c.2921G>A, leading to the substitution p.Gly974Glu (G974E) (rs1201230394), have been analyzed. The analysis with the in silico server was based on the evolutionary conservation of the mutated site and on the type of variant. Both variants involve highly conserved amino acids and are, therefore, probably pathogenic, especially the first one. For the second, the response is more moderate and variable (for more in-depth results, see Supplementary Files S3 and S4).

**Figure 2:**
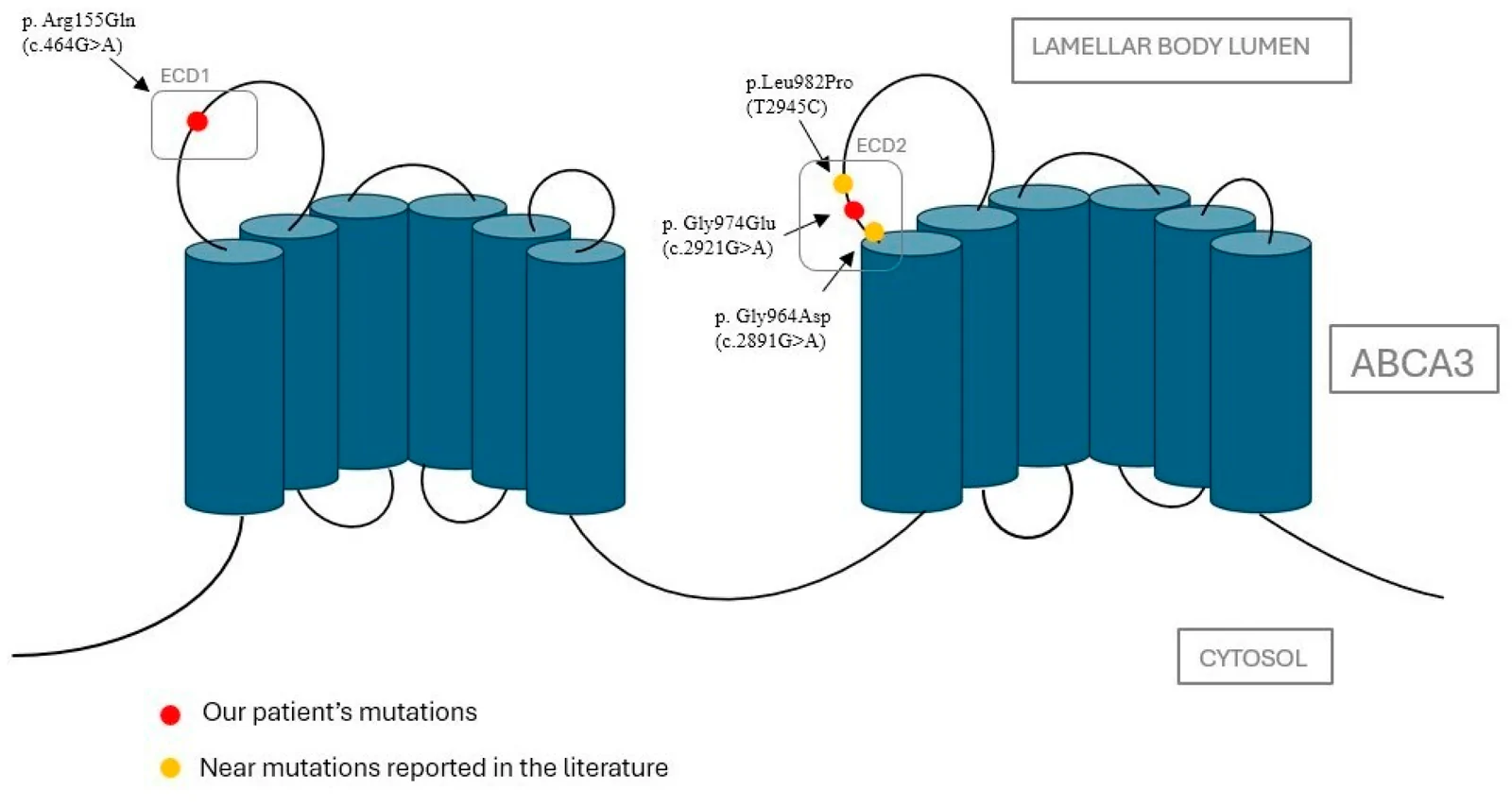
(Modified by Beers MF et al., 2016 [18]). A 3D reconstruction of the ABCA3 protein in its cellular context. The first variant, reported as pathogenic, is clearly shown within the ECD n.1 (**left**), as R155Q. The second one (G974E), never described to date, is located in the context of ECD n.2 (**right**), very close to two variants (G964S and L982P) already known in the literature and leading both to pathogenic effects, as described in the Section 3.

A recent study by Beers et al. [18] analyzed in detail the known variants of the *ABCA3* gene. Figure 2 maps the current variants on the reconstructed 3D structure in its cellular context, showing that they are both located in the extracellular domains (ECDs), which are inside the lumen of the lamellar bodies (LBs).

## 3. Discussion

Different causes of interstitial lung disease may be recognized. The case herein described falls within the chILD subtype ‘surfactant dysfunction’. Actually, congenital surfactant protein deficits represent a significant cause of interstitial disease. The proteins mainly involved are surfactant protein C (SP-C), surfactant protein B (SP-B), and ABCA3. The ABCA3 protein plays a role in the transport of phosphatidylcholine and phosphatidylglycerol into the lamellar bodies, where the final processing of surfactant components prior to secretion takes place [19]. Abnormalities in this protein cause the altered transport of lipids that become trapped in the endoplasmic reticulum, triggering apoptosis in alveolar epithelial cells and the initiation of fibrogenesis [20]. Its deficiency is transmitted with autosomal recessive inheritance.

Besides presenting the clinical case of a newborn girl affected by a novel compound heterozygous variant of the *ABCA3* gene, we also carried out a mini-review of the recent available literature (2004–2025) on similar pediatric cases, highlighting similarities and differences. To this aim, English reviews and case reports were included. We excluded letters and series. The following keywords (alone or in combination) were used: chILDs, newborns, interstitial disease, respiratory insufficiency, surfactant, pediatric, ABCA3, genetic, diagnosis, therapy. The electronic databases used were PubMed and Scopus. The cases described in the literature who carry both the homozygous and compound heterozygous variants are summarized in Table 1.

Our patient carried a compound heterozygous variant of *ABCA3*, and according to the literature reports, the prognosis seems to be better than that observed in subjects with homozygous variants. The clinical implications, however, also depend on the type of variant. The extent of the variation in the case of a deletion, or the site where it falls in the case of missense, nonsense, or frameshift ones, can modify the functionality of the protein, up to its complete loss. In the specific case of our patient’s variant, it was found that it falls into highly conserved amino acids and, therefore, may be considered probably pathogenic. The G974E variant described in our patient has never been reported in the literature, but a pathogenetic role can be assumed for it. Actually, it is located close to two already known variants: L982P and G964D. In particular, with regard to L982P, two patients carrying this variant in compound heterozygosity had an early fatal outcome, occurring during the neonatal period and at three months of age, respectively [42]. The G964D variant has been analyzed by Schindlbeck et al. [43], but its role has not been well defined. It shows discrete functionality in vitro, and Campo et al. described in 2014, indeed, the case of a girl carrying such a variant and diagnosed with pulmonary fibrosis in adulthood, leading to suggest for it a less severe pathogenic role [43,44]. Clinical pictures caused by the *ABCA3* variant may also differ in severity within siblings with the same genotype [23,28]. It is likely that, in addition to genetic factors, epigenetic or environmental factors may also contribute to the severity of the respiratory picture. Moreover, a higher prevalence and mortality rate has been observed among male subjects. Actually, premature males have a higher incidence of respiratory distress syndrome (RDS) and bronchopulmonary dysplasia, as androgens are able to delay the maturation of the alveolar epithelium, and they act as positive regulators in the pathway involved in pulmonary fibrosis. In contrast, estrogens allow the promotion of the maturation of the alveolar epithelium and increase the presence of proteins in the surfactant [28]. All such aspects together might explain a mild evolution of the lung impairment observed in our baby.

Surfactant deficiency from ABCA3 protein alterations typically manifests with neonatal respiratory failure [22], as also occurred in the present patient. In the literature, however, cases with late onset (i.e., 9 months, 8 years) [31,41] or with variable manifestations (pulmonary hypertension, chronic cough) [21,41] have also been reported. Neonatal respiratory distress is a clinical condition that generally affects preterm infants due to lung immaturity, the poor development of alveolar type II cells, and, consequently, reduced surfactant production. Its incidence is greater the younger the infant’s gestational age at birth. The presence and/or persistence of symptoms in full-term or late-preterm babies can, therefore, be considered as a warning for the potential subtending occurrence of a congenital surfactant deficiency. Our patient, like several cases described in the literature [24,36], is a late preterm who had a persistent need for respiratory support. This aspect, along with the difficulty in ventilation weaning and the exclusion of other causes, led us to suspect that RDS was not linked to prematurity but rather to a congenital lung disease [30,40]. Furthermore, the absent or transient response to exogenous surfactant administration, as observed in our case and similarly reported in previous studies of the literature, represents a further suggestive element that must guide clinicians towards the suspicion of a congenital surfactant deficiency.

Chai et al. highlighted that ABCA3 protein deficiency may lead to lipid accumulation in the endoplasmic reticulum, resulting in the apoptosis of alveolar cells and the production of pro-inflammatory cytokines [19]. Furthermore, Rindler et al., in a recent study of 2017, documented and proved an anti-inflammatory function of the ABCA3 protein [45]. It has also been hypothesized that the protein defect leads to a reduction in the secretion of surfactant proteins (such as the hydrophilic collectins SP-A and SP-D), which play an immunomodulatory role by enhancing the phagocytosis of apoptotic cells and inhibiting T-cell function [19]. All these findings may explain the high susceptibility and harm experienced by such patients when facing infections, also in light of the antimicrobial role played by surfactants (in our case, indeed, two life-threatening bacterial infections were reported). To date, the only effective treatment option for these patients is lung transplantation. The operation is not free from both early and late complications, such as bleeding, infection, early rejection, bronchiolitis obliterans, and restrictive allograft syndrome. The 5-year survival rate for lung transplants remains approximately 60% for infants and 80% for children. Other therapeutic approaches are based on corticosteroids, hydroxychloroquine, or azathioprine, which can positively influence the prognosis. Not all children respond to these therapies, however, and this probably depends on the genetic defect and the type of variant. The exact mechanism of action of hydroxychloroquine is unknown, but it appears to reduce interstitial inflammation. In vitro, this drug showed efficacy, especially for the E292V, D953H, Q1045R, and A1046E variants, by increasing lipid transport [46]. Corticosteroids appear to regulate ABCA3 expression in the alveolar type II cells. Macrolides are able to inhibit the production of inflammatory cytokines and mediators involved in pulmonary fibrosis [27]. According to the above literature, survival was higher among patients receiving such triple therapy, and its benefits have also been proven in our experience. Some of the surviving children reported to date needed persistent oxygen support and showed growth impairment, in addition to neuromotor, linguistic, and cognitive delay [23,30,33,37,40,41]. In our case, the baby is dependent on high-flow oxygen therapy, while gastrostomy is, at present, able to ensure sufficient growth. No neurological, cognitive, or other developmental issues are currently noticed, except for mild motor delay. In the present case, therapy was administered according to the experimental protocol for childhood interstitial lung disease [6]. The treatment allowed a significant improvement in the patient’s condition and, finally, her discharge after around 5 months. In addition to the pharmacological approaches already analyzed, new frontiers of research are turning their gaze towards gene therapy, whereby DNA fragments or mRNA are carried by viral vectors (most frequently adenoviruses) or nanoparticles that, unlike viruses, do not trigger an immune system response [46]. Pulmonary interstitial diseases are chronic pathologies that require continuous care. It is, indeed, essential to educate parents so that they can be prepared for the home management of their children but also to provide them with medical and nursing support through the health territory services. Home management, in our experience, is provided through a complex home care service by the local health authority that is integrated with the activities of the healthcare workers of our department, who guarantee the family the necessary tools and devices to manage and protect their child.

## 4. Conclusions

ChILDs are a rare cause of congenital lung disease, which includes, among others, congenital surfactant deficiency disorders. They can have different age presentations but generally occur in the neonatal period with the onset of respiratory distress, completely or partially unresponsive to the administration of an exogenous surfactant. Knowing the genetic profile of these patients has a crucial role, as for other rare diseases, due to its relevant implications for diagnosis, as well as for the therapeutic approach and prognosis evaluation [47,48,49]. To date, the only curative treatment option for these patients is lung transplantation, although the use of corticosteroids, azathioprine, and hydroxychloroquine has shown a favorable effect on both prognosis and survival of affected subjects. Drugs capable of increasing the functionality or expression of the mutated proteins (i.e., ABCA3), antifibrotic drugs (already used in adult patients with pulmonary interstitial disease), and, above all, gene therapy (using viral vectors in the case of surfactant protein variants) may allow the development of targeted treatments for congenital surfactant protein deficiency. An integrated development of these pharmacological approaches may eventually improve the survival of patients and the quality of life for them and their whole families in the future.

## Figures and Tables

**Table 1:** Comparison between our patient and those previously described in the literature affected by surfactant dysfunction due to *ABCA3* variants. GA: gestational age. RDS: respiratory distress syndrome. HFNC: high-flow nasal cannula.

Authors, Year, Country	Type of Mutation in the *ABCA3* Gene	Sex	GA	Onset	Clinical Features	Surfactant	Treatment	Outcome
Kuning et al. [21] 2007, Colorado (USA)	p.Leu326Arg, in homozygosity	M	Term	Early onset	Refractory pulmonary hypertension, later parenchymal lung disease	No (due to clinical instability)	Nitric oxide, dopamine (for pulmonary hypertension)	Death due to withdrawal of intensive care
Anandarajan et al. [22] 2009, United Kingdom	p.Gly378Arg, p.Gly1002Arg	F	Term	Within few hours of birth	RDS	Yes	Antibiotics (not specified)	Death at 25 days of life due to withdrawal of intensive care
Ciantelli et al. [9] 2011, Italy	p.Pro186Thr, p.Met878Trp fs36X	M	Term	Within few hours of birth	RDS	Yes (9 doses)	Dexamethasone	Death at 72 days of life
Hallik et al. [23] 2013, Estonia	p.Asp507AlafsTer508, p.Asp696Asn	F	Term	Within few hours of birth	RDS	Yes (1 dose)	Methylprednisolone, azithromycin, hydroxychloroquine	Discharged at age 12 months with oxygen therapy and gastrostomy. In the following months, mild neuro-motor delay.
		M	Not specified					Asymptomatic for 4 years
Goncalves et al. [24] 2014, Portugal	p.Leu798Pro, p.Arg1612Pro	F	Term	Within few hours of birth	RDS	Yes (4 doses)	Methylprednisolone, hydroxychloroquine	Death at 101 days due to withdrawal of intensive care
Maly et al. [25] 2014, Czech Republic	p. Met1227Arg, p. Ins1510fsTer1519	M	Term	Within few hours of birth	RDS	Yes	Dexamethasone and macrolides	Death at 33 days of life due to withdrawal of intensive care
Moore et al. [26] 2014, Canada	p.Ser1116Phe, in homozygosity	M	Term	At three hours of life	RDS	Yes (a few doses)	Antibiotics, nitric oxide	Death at 48 days of life
		F	Term	At sixteen hours of life	RDS	Yes		Death at 53 days of life
Jackson et al. [27] 2015, USA	p.Arg280Cys, p.Val1399Met, p.Gln1589Ter	F	Term	Within few hours of birth	RDS	Yes	Nitric oxide	Bilateral lung transplant at 9 months. At age 3 years, without oxygen support, nutritional supplementation tube, mild motor and speech delay.
Piersigilli et al. [28] 2015, Italy	p.Arg194Gly, p.Val1615GlyfsTer15	F	Late preterm	Within few hours of birth	RDS	Yes (3 doses)	Betamethasone, hydroxychloroquine	Death at age 5 months
		M		Early onset	RDS	Yes (three doses)	Betamethasone, hydroxychloroquine	Death at age 3 months
Pachajoa et al. [29] 2016, Colombia	IVS 25-98T in homozygosity	M	Term	Early onset	RDS	Yes	Antibiotics (vancomycin, meropenem)	Death at 60 days
Tan et al. [30] 2016, Australia	p.Ala307Val, in homozygosity	F	Preterm	Early onset	RDS	Yes (2 doses)	Hydroxychloroquine, azithromycin, methylprednisolone	Oxygen therapy through nasal cannula at age 1 year
Ota et al. [31] 2016, Japan	p.Leu34Pro, p.1203_1204del	F	Term	8 years	Pulmonary fibrosis with emphysema, pulmonary hypertension	Not specified	Calcium antagonist, warfarin, home oxygen therapy, beraprost sodium	Improvement of pulmonary arterial pressure. No worsening of fibrosis.
Uchida et al. [32] 2017, USA	p.(delPhe1203)4, c.1375ins15	F	Term	Early onset	RDS, perihilar and peripheral rounded masses of the right lung	Not specified	Systemic corticosteroids (not specified)	Death at 113 days
Akil et al. [33] 2018, USA	p.Glu292Val, p.Met1647fs	M	Term	Early onset	Respiratory illness, cyanosis, impaired growth.	Not specified	Prednisolone, methylprednisolone, azithromycin, hydroxychloroquine. Gastrostomy	Oxygen therapy with nasal cannula at age 1 year
Mitsiakos et al. [34] 2019, Greece	p.Asp1149Asn, in homozygosity	M	Term	Within few hours of birth	RDS	Yes (10 doses)	Methylprednisolone, prednisolone, azithromycin. At age 78 days hydroxychloroquine, prednisone, azithromycin	Death at 9 months
Oltvai et al. [35] 2020, USA	p.Ala1629GlyfsX15, p.Gly961Gly	M	Term	Within few hours of birth	RDS	Yes (a few doses)	Dexamethasone and macrolides	Successful bilateral lung transplantation at 15 weeks of age
Gupta et al. [36] 2020, India	p.Leu437Pro, in homozygosity	F	Late preterm	Within few hours of birth	RDS	Yes (2 doses)	Dexamethasone	Death at 100 days
Shaaban et al. [37] 2021, Kuwait	c.875A<T in homozygosity	M	Term	Within few hours of birth	RDS	Yes	Hydroxychloroquine, methylprednisolone, azithromycin	Discharged at 160 days with hydroxychloroquine maintenance treatment
Bozkurt et al. [38] 2021, Turkey	p.Leu1226Pro, in homozygosity	M	Term	Early onset	RDS, pulmonary hypertension	Yes (3 doses)	Antibiotics, nitric oxide, sildenafil	Death at 24 days
Zhang et al. [39] 2021, China	c.4-7del, p.Lys291Lys	Two girls	Term	Within few hours of birth	RDS	Yes	Antibiotics, nitric oxide, and methylprednisolone	Death at 23 days
Si et al. [40] 2021, California (USA)	p.Glu292Val, p.Arg1081Trp	M	Pre term	Early onset	RDS	Yes	Azithromycin, hydroxychloroquine, methylprednisolone. Gastrostomy *fundoplicatio* according to Nissen	At age 2 years without oxygen support, severe motor, language and cognitive delay, impaired growth. Steroid therapy discontinued, bilevel alternating with low-flow nasal cannula respiratory support
	p.Tyr758Ter, p.Lys915Asn	M	Term	Within few hours of birth	RDS	Yes	Methylprednisolone, hydroxychloroquine, azithromycin. Gastrostomy *fundoplicatio* according to Nissen	At age 14 months, language, cognitive, and motor delay, impaired growth. Steroid discontinued at 1 year. HFNC respiratory support.
	C.1285+1G>A, p.Asp200Gly	M	Term	Within few hours of birth	RDS	Yes	Methylprednisolone, azithromycin and hydroxychloroquine. Gastrostomy fundoplicatio according to Nissen	Removal of gastrostomy tube at age 18 months, impaired growth, mild language delay (at age 13 months). Steroid discontinued. Low-flow nasal cannula (LFNC).
Chen et al. [41] 2022, China	c.4035+5G>A, p.Met223Thr, c.1285+4A>C	M	Term	After 24 h of birth	Dyspnea and cough	Not specified	Azithromycin, dexamethasone and hydroxychloroquine	Death at 94 days
	p.Phe235Ser, p.Thr1346Asn fsTer15	M	Term	At 9 months	Chronic cough	Not specified	Budesonide, ipratropium bromide and acetylcysteine	At age 41 months, stable clinical condition without respiratory support
Our case (2025)	p.Arg155Gln, p.Gly974Glu	F	Late preterm	Early onset	RDS	Yes (4 doses)	Azithromycin, hydroxychloroquine, diuretics (furosemide, spironolactone), sildenafil. Gastrostomy	Good general condition with oxygen therapy through HFNC. Impaired growth, mild motor delay.

## Data Availability

The original contributions presented in this study are included in the article and Supplementary Materials. Further inquiries can be directed to the corresponding author and/or to the first author.

## Supplementary Materials

The following supporting information can be downloaded at: https://www.mdpi.com/article/10.3390/jcm14113704/s1, Supplementary File S1. Panel of the genes included within the current next-generation sequencing analysis. Supplementary File S2. Prenatal genetic investigations performed on chorionic villi sample during the second pregnancy of the proband’s mother. Supplementary File S3. Bioinformatic analysis of the identified variants, through SIFT, Poly-Phen-2, MutationTaster, and Panther tools. Supplementary File S4. Detail of the amino acid adjacent to each substitution and probabilities of protein function defect according to SIFT.
