# Supplementary material for: Novel Compound Heterozygous Mutation of the ABCA3 Gene in a Patient with Neonatal-Onset Interstitial Lung Disease
Source: J Clin Med. 2025 May 25;14(11):3704. doi: 10.3390/jcm14113704 (PMC12155473; doi:10.3390/jcm14113704)
Supplement: Supplementary file 1 [file jcm-14-03704-s001.zip › Supplementary file 3.pdf]

Supplementary file 3, S3. Bioinformatic analysis of the identified variants, through SIFT, Poly-Phen-2, MutationTaster, and Panther tools

According to SIFT predictions, substitution at position 155 from R to Q is predicted to affect protein function with a score of 0.00, median sequence conservation: 3.33, sequences represented at this position:7. Conversely, substitution at position 794 from G to E is predicted to affect protein function with a score of 0.00, median sequence conservation: 3.10, sequences represented at this position:11 (details about the amino acids adjacent to each substitution are presented in Supplementary file 4, S4). Based on Mutation Taster, the first alteration has been predicted to be disease causing (Model, simple_aae, prob: 0.999998555252059), while the second one as a polymorphism (Model, simple_aae, prob: 0.999999997138398). Moreover, Panther classification system found for the first substitution a preservation time of 1237, and a probability of deleterious effect (Pdel) of 0.85, leading to consider it as probably damaging; while, for the second variant, a preservation time of 220, with a Pdel of 0.5 and a possibly damaging effect was detected. In accordance with such evolutionary analysis of coding SNPS, PSEP (position-specific evolutionary preservation) measures the length of time (in millions of years) a position in current protein has been preserved by tracing back to its reconstructed direct ancestors. The longer a position has been preserved, the more likely that it will have a deleterious effect. The system converts this to a probability of deleterious effect (Pdel) from results on the HumVar benchmark. This Pdel is converted into a qualitative prediction as follows: "probably damaging" (time > 450 my, corresponding to a false positive rate of ~0.2 as tested on HumVar), "possibly damaging" (450 > time > 200my, corresponding to a false positive rate of ~0.4), and "probably benign" (time < 200 my). Finally, Polyphen-2 revealed for the first variant the prediction to be probably damaging, according to both HumDir and HumVar (with a score of 1.000 for both; sensitivity 0.00, specificity 1.00); the second substitution, instead, was predicted to be possibly damaging or benign, according to HumDir and HumVar, with a score of 0.494 and 0.236, respectively (sensitivity 0.88, specificity 0.90 for the former tool; sensitivity 0.88, specificity 0.75 for the latter).
