# Supplementary material for: Novel Compound Heterozygous Mutation of the ABCA3 Gene in a Patient with Neonatal-Onset Interstitial Lung Disease
Source: J Clin Med. 2025 May 25;14(11):3704. doi: 10.3390/jcm14113704 (PMC12155473; doi:10.3390/jcm14113704)
Supplement: Supplementary file 1 [file jcm-14-03704-s001.zip › Supplementary file 4.pdf]

Supplementary file 4, S4. Detail of the amino acid adjacent to each substitution, and probabilities of protein function defect according to SIFT

Each row corresponds to a position in the reference protein. Below each position is the fraction of sequences that contain one of the basic amino acids. A low fraction indicates the position is either severely gapped or unalignable and has little information. Expect poor prediction at these positions.

Each column corresponds to one of the twenty amino acids.

Each entry contains the score at a particular position (row) for an amino acid substitution (column). Substitutions predicted to be intolerant are highlighted in red.

First substitution c.464G>A, leading to p.Arg155Gln (R155Q):

	pos	A	C	D	E	F	G	H	I	K	L	M	N	P	Q	R	S	T	V	W	Y
**151K**	**0.64**	0.44	0.05	0.32	0.55	0.12	0.27	0.22	0.16	1.00	0.28	0.10	0.35	0.23	0.46	0.73	0.45	0.44	0.22	0.04	0.18
**152Y**	**0.64**	0.65	0.14	0.51	0.30	0.48	0.73	0.31	0.21	0.33	0.37	0.12	0.60	0.26	0.24	0.28	0.55	0.33	0.30	0.13	1.00
**153H**	**0.64**	0.28	0.04	0.20	0.34	0.10	0.21	0.40	0.12	1.00	0.21	0.07	0.28	0.16	0.36	0.72	0.31	0.39	0.16	0.04	0.16
**154L**	**0.64**	0.03	0.01	0.00	0.01	0.06	0.01	0.00	0.71	0.01	1.00	0.07	0.01	0.01	0.01	0.01	0.01	0.02	0.16	0.01	0.01
**155R**	**0.64**	0.00	0.00	0.00	0.00	0.00	0.00	0.00	0.00	0.00	0.00	0.00	0.00	0.00	0.00	1.00	0.00	0.00	0.00	0.00	0.00
**156F**	**0.36**	0.08	0.02	0.02	0.03	1.00	0.03	0.03	0.21	0.03	0.30	0.07	0.03	0.03	0.03	0.03	0.04	0.06	0.37	0.05	0.19
**157S**	**0.36**	0.41	0.04	0.31	0.41	0.05	0.26	0.10	0.09	0.83	0.15	0.06	0.31	0.17	0.28	0.36	1.00	0.42	0.15	0.02	0.06
**158Y**	**0.36**	0.20	0.07	0.07	0.10	0.63	0.11	0.16	0.73	0.11	0.52	0.13	0.10	0.08	0.10	0.12	0.15	0.16	0.53	0.14	1.00
**159T**	**0.36**	0.53	0.07	0.30	0.48	0.10	0.28	0.18	0.19	1.00	0.29	0.10	0.38	0.25	0.45	0.81	0.74	0.92	0.30	0.04	0.14
**160R**	**0.36**	0.32	0.05	0.13	0.30	0.15	0.18	0.16	0.22	0.86	0.48	0.30	0.20	0.15	0.35	1.00	0.25	0.26	0.25	0.04	0.16

Second substitution c.2921G>A, leading to p.Gly974Glu (G974E):

**972V**	**0.64**	0.90	0.13	0.18	0.29	0.15	0.28	0.11	0.46	0.28	0.43	0.17	0.18	0.23	0.20	0.19	0.53	0.53	1.00	0.04	0.15
**973P**	**0.64**	0.13	0.01	0.03	0.03	0.01	0.07	0.01	0.01	0.03	0.02	0.01	0.04	1.00	0.02	0.02	0.53	0.08	0.03	0.00	0.01
**974G**	**0.64**	0.00	0.00	0.00	0.00	0.00	1.00	0.00	0.00	0.00	0.00	0.00	0.00	0.00	0.00	0.00	0.00	0.00	0.00	0.00	0.00
**975T**	**0.64**	0.58	0.07	0.97	0.74	0.06	0.65	0.20	0.13	0.69	0.21	0.08	1.00	0.27	0.45	0.39	0.87	0.68	0.20	0.03	0.10
