# Supplementary material for: Novel Compound Heterozygous Mutation of the ABCA3 Gene in a Patient with Neonatal-Onset Interstitial Lung Disease
Source: J Clin Med. 2025 May 25;14(11):3704. doi: 10.3390/jcm14113704 (PMC12155473; doi:10.3390/jcm14113704)
Supplement: Supplementary file 1 [file jcm-14-03704-s001.zip › Supplementary file S1 11.5.25.pdf]

Supplementary file 1, S1. Panel of the genes included within the current next generation sequencing analysis.

Gene	RefSeq
FOXF1	NM_001451
SFTPA1	NM_005411
SFTPA2	NM_00109866
ACVRL1	NM_000020
BMPR2	NM_001204
CBLN2	NM_182511
CRHR1	NM_ 001145146
ENG	NM_000118
SMAD9	NM_005905
TBX4	NM_018488
SMAD1	NM_005900
PPARG	NM_015869
MEOX2	NM_005924
CSF2RB	NM_000395
CSF2RA	NM_172245.4
NKX2-1	NM_001079668.2
SMAD5	NM_005903
ABCA3	**NM_001089**
SFTPC	NM_003018
SFTPB	NM_000542
SFTPD	NM_003019
SLC7A7	NM_001126106
MARS1	NM_004990.4
GATA2	NM_032638.5
OAS1	NM_016816.4
KCNK3	NM_002246

CAV1	NM_001753
EIF2AK4	NM_001013703
SLC34A2	NM_006424.2
MUC5B	NM_002458.3
FLNA	NM_001110556.2
