# Supplementary material for: Novel Compound Heterozygous Mutation of the ABCA3 Gene in a Patient with Neonatal-Onset Interstitial Lung Disease
Source: J Clin Med. 2025 May 25;14(11):3704. doi: 10.3390/jcm14113704 (PMC12155473; doi:10.3390/jcm14113704)
Supplement: Supplementary file 1 [file jcm-14-03704-s001.zip › Supplementary file S2.pdf]

Supplementary file 2, S2. Prenatal genetic investigations performed on chorionic villi sample during the second pregnancy of the proband's mother

Next generation sequencing analysis to search for the familial variants of the *ABCA3* gene (NM_001089.3) has been performed on chorionic villi sample within the first trimester of the second gestation of proband's mother. DNA was extracted from the biological sample. Assays for the exclusion of maternal contamination have been carried out. The sequencing used allowed to simultaneously analyze millions of small sequences of the gene under investigation, assessing the presence of mutations, small insertions and/or deletions. The platform applied was NextSeq550DX Illumina; the kit employed was ClinEX pro kit (4bases). Bioinformatic analysis for the identification of eventual mutations in the considered gene has been conducted through the BWA Aligner or DRAGEN Germline Pipeline systems, and the sequences were aligned to the reference human genome GRCh37. Geneyx Analysis software (Knowledge-Driven NGS Analysis tool powered by the GeneCards Suite) was used for filtering and prioritizing variants. Updated databases Human Gene Mutation Database (www.hgmd.org), ClinVar (http://www.ncbi.nlm.nih.gov/clinvar/), Varsome (http://varsome.com/), and dsSNP (http://www.ncbi.nlm.nih.gov/project/SNP) were considered to interpret the results. Variants were annotated according to the HGVS nomenclature, and classified according to the ACMG Standard Guidelines. Average test coverage: 200X. Average genetic coverage: >99%. Analytical sensitivity and specificity: >99%. Limitations of the test: the presence of any homologous chromosomal regions (pseudogenes), with high sequence similarity, decreases the sensitivity and specificity of the analysis. Furthermore, the test cannot identify single exon duplications and deletions, multiexonic rearrangements or those involving the entire gene, complex genomic rearrangements, and mutations due to expansion of repeated sequences (dynamic mutations) which can be characterized with other analytical methods. Finally, the test also has a limited resolution in the identification of mosaicisms. Molecular analysis of the familial variants c.464G>A (p.Arg155Gln) and c.2921G>A (p.Gly974Glu) of the *ABCA3* gene (NM_001089.3) performed through the method above described on the DNA extracted from the sample under examination (chorionic villi), detected in the *ABCA3* gene in homozygous state, the presence of the only variant c.464G>A, which at the protein level determines the amino acid change Arg155Gln. This missense mutation, segregated by the heterozygous mother, is not present in the Database of allele frequencies of the general population (gnomAD), and can be classified according to the ACMG guidelines as probably pathogenic (class 4). Moreover, the presence of the c.2921G>A (p.Gly974Glu) variant was not detected. In light of the NGS analysis results, the DNA polymorphisms (Microsatellites - STR -) test related to chromosome 16 was carried out. DNA extraction was then performed through routine methods on the chorionic villus sample under investigation. Assays to exclude maternal contamination have been conducted. The DNA was amplified by PCR, and the fragments were analyzed by capillary electrophoresis of the following Short Tandem Repeats (STR) located on chromosome 16: D16S521, D16S446, D16S3102, D16S3079, D16S403, D16S770, D16S3112, D16S3096, D16S3104. The resolution of the amplified products was obtained by Fluorescent Capillary Electrophoresis, using the automatic sequencer SeqStudio Genetic Analyzer (PE Applied Biosystems). Finally, the allelic patterns have been defined, and a comparison of the genetic profiles obtained was made. The test performed had sensitivity of 99.00%, and a limited resolution in identifying any mosaicisms. The analysis of the STR polymorphic markers finally identified a maternal uniparental disomy of chromosome 16, explaining thus the genomic profile identified in the previous NGS investigation (the homozygous c.464G>A variant in the *ABCA3* gene).
